# Genetic and Pathogenicity Studies of Avian Influenza (H9N2) Virus Isolated from Chickens in Jiangsu Province, China, in 2025

**DOI:** 10.3390/microorganisms14081667

**Published:** 2026-07-30

**Authors:** Yue Li, Xingdong Song, Tengfei Gao, Guangyuan Wang, Hongyin Wei, Jiajun Wang, Wenjun Jiang, Ruihua Zhang, Yu Meng, Shijin Jiang

**Affiliations:** 1 Department of Preventive Veterinary Medicine, College of Veterinary Medicine, Shandong Agricultural University, Taian 271017, China; liyue1681217@163.com (Y.L.); songxingdong05@163.com (X.S.); 17662589503@163.com (T.G.); 17685747152@163.com (G.W.); 15610336726@163.com (H.W.); 18562318325@163.com (J.W.); jwj6596@163.com (W.J.); rhzhang@sdau.edu.cn (R.Z.); 2 Shandong Provincial Key Laboratory of Zoonoses, Taian 271017, China

**Keywords:** avian influenza virus, H9N2 subtype, chicken-derived, phylogeny, pathogenicity

## Abstract

In this study, one H9N2 subtype avian influenza virus (AIV) was isolated in Jiangsu Province, China, in March 2025. According to phylogenetic analysis, the HA gene fragment of the H9N2 isolate was classified as the B4.7 lineage, with the remaining seven segments falling into the Eurasian lineage. This H9N2 virus underwent complex genetic reassortment across multiple regions in China during its evolution, and key amino acid substitutions were identified, which may enhance its viral polymerase activity and replication efficiency in mice. Critically, a mouse experiment demonstrated that the H9N2 virus replicated effectively in the lungs and nasal turbinates of BALB/c mice without requiring prior adaptation. In addition, poultry infection studies showed that the H9N2 virus could replicate and spread more efficiently in chickens than in ducks. Moreover, the virus could be transmitted to contacted ducks through infected chickens with greater efficiency than from ducks to ducks. Furthermore, in the lungs of inoculated chickens and ducks, the mRNA expression levels of pattern-recognition receptors (PRRs) and cytokines were upregulated, while the overall fold increase in ducks was lower than that in chickens. In conclusion, these results highlighted the potential public health risk presented by chicken-derived H9N2 AIV, emphasizing the critical need for enhanced routine surveillance of H9N2 AIVs from poultry.

## 1. Introduction

Avian influenza virus (AIV) is a member of the *type A influenza virus* genus in the *Orthomyxoviridae* family and is an enveloped, negative-sensed, segmented single-stranded RNA virus [1]. According to their surface hemagglutinin (HA; 18 subtypes) and neuraminidase (NA; 11 subtypes) glycoproteins, influenza A viruses (IAVs) can be classified into different subtypes [2]. All known HA and NA subtypes, except for H17N10 and H18N11 isolated from bats, have been identified in wild aquatic avian species [3,4]. In addition, AIV can be further categorized as Highly pathogenic avian influenza virus (HPAIV) or Low pathogenic avian influenza virus (LPAIV) based on either the Intravenous pathogenicity index (IVPI) of chicken-tested viruses or the existence of a polybasic cleavage site in HA [5,6]. Outbreaks of HPAI have caused enormous damage to poultry farming and presented significant risks to human well-being [7,8]. Although LPAIV is less pathogenic than HPAIV, its potential risk to public health security cannot be underestimated [9,10]. For instance, from 1999 to 2023, human infections with AIVs of H9N2, H6N1, H10N3, H3N8, and H10N5 were documented in China [11,12,13,14,15].

In 1966, H9N2 AIV was initially detected in turkeys in the state of Wisconsin in the United States [16]. Subsequently, it has become endemic across numerous countries in Africa, Asia, and Europe [17,18]. Poultry, particularly chickens, are the primary hosts of the H9N2 virus [19]. With evidence supporting viral transmission to ducks from chickens [20], however, the extent and mechanisms of this cross-species spread remain unclear, providing a rationale for our study. In addition, these viruses have the potential to cross species boundaries to infect mammals [10,21]. H9N2 viruses have been reported to be isolated from pigs, weasels, dogs, minks, and even human beings [22,23,24]. Following the first known human case of H9N2 virus infection in Hong Kong in 1999 [25], subsequent human infections were identified in multiple countries [26,27]. In addition, serological evidence of human infection was reported across Africa, Asia, North America, and the Middle East from 1997 to 2013 [28]. More recently, additional human cases of H9N2 infection continued to be documented from 2015 to 2025 [29,30,31]. Moreover, H9N2 viruses could contribute their partial or even complete internal genes to fatal AIVs, such as H7N9, H5N2, H10N8 and H5N6 AIVs, presenting a considerable public health hazard [32,33,34,35,36,37]. These facts prompted us to evaluate the risk of cross-species infection posed by H9N2 AIVs.

In March 2025, we isolated one chicken-derived H9N2 virus in Xuzhou City, Jiangsu Province, China. A search of the GISAID database (http://platform.gisaid.org) revealed that over the past three years only two B4.7 lineage H9N2 AIVs with full-genome sequences from Jiangsu Province, namely, A/Suzhou/1209KS/2025 and A/chicken/Jiangsu/M2342C45/2023, have been publicly deposited, and our isolate exhibited considerable amino acid divergence from these two viruses, with 91 and 90 distinct sites, respectively. Given this divergence, we investigated its genetic relationships, gene reassortment, molecular characteristics, and pathogenicity in chickens, ducks and mice. Our data showed that the H9N2 AIV isolated from chickens poses a potential public health threat, and it is essential to intensify routine monitoring of H9N2 AIVs in poultry.

## 2. Materials and Methods

### 2.1. Virus Isolation, RNA Extraction and Genetic Identification

Between September 2024 and December 2025, a total of 326 samples (including oral swabs, cloacal swabs, trachea tissue specimens and lung tissue specimens from poultry) were collected from eight Chinese provinces: Shandong (*n* = 262), Jiangsu (*n* = 23), Henan (*n* = 13), Hebei (*n* = 10), Guangdong (*n* = 7), Jilin (*n* = 6), Heilongjiang (*n* = 3), and Anhui (*n* = 2). After being vortexed and centrifuged, the samples were used to inoculate specific-pathogen-free (SPF) 11-day-old chicken embryos. Live H9N2 virus experiments and virus isolation took place in a biosafety level 2 laboratory at Shandong Agricultural University (SDAU). The allantoic fluid was obtained after incubation at 37 °C for 72 h and was then tested for hemagglutination activity using 1% chicken red blood cells. The hemagglutination-positive samples were used to obtain total viral RNA with an RNA extraction kit (Tiangen, Beijing, China), and cDNA was generated from the extracted RNA by utilizing a reverse transcription kit (Yugong Biotech, Lianyungang, China) in a 20 μL reaction system containing 1 μg total RNA, 4 μL Synthesis MasterMix, 1 μL dsDNase, and Nuclease-Free Water. The reaction mixture was incubated at 37 °C for 2 min, 55 °C for 15 min, and 85 °C for 5 min. This method is applicable to all RNA types. The eight AIV gene segments underwent RT-PCR amplification with specific primers. Subsequently, the PCR products were sent to Sangon Biotech (Jinan, Shandong Province, China) after purification for Sanger sequencing. The obtained sequences were assembled using SeqMan Pro (DNASTAR Lasergene 11.0). All primer sequences used in this study were obtained from a previous study [38].

### 2.2. Phylogenetic Analysis

HA gene representatives were chosen from each major clade (G1, B1–B3, and B4.1–B4.7) following the established H9 subtype classification system [39]. The remaining seven gene segments were analyzed by BLAST against the NCBI influenza virus database (http://www.ncbi.nlm.nih.gov/genomes/FLU/FLU.html, accessed on 30 May 2025) and the GISAID database, and reference sequences from the North American lineage were also included to maximize phylogenetic diversity. Phylogenetic analysis of each gene fragment was conducted by the maximum likelihood (ML) method, with a GTR + G + I nucleotide substitution model and 1000 bootstrap replicates, and the software used was MEGA 11.0, as described in a previous study [40]. A 97% sequence identity cut-off was applied for the classification of gene fragments in all of the phylogenetic trees [41].

### 2.3. Pathogenicity and Transmission Experiments in Chickens and Ducks

A total of 100 3-week-old SPF chickens (Boehringer Ingelheim Vital Biotechnology, Beijing, China) and 100 3-week-old SPF ducks (Harbin Veterinary Research Institute, Harbin, China) were each separated into one inoculation group (*n* = 50) and one control group (*n* = 38). Each inoculation group was intranasally inoculated with 100 μL of 10^6^ 50% embryo infective dose (EID_50_) of H9N2 AIV, whereas control animals were dosed with 100 μL of phosphate-buffered saline (PBS). At 24 h post-infection (hpi), 12 uninfected chickens and 12 uninfected ducks were designated as contacted groups and co-housed with inoculated chickens and inoculated ducks, respectively. At 3, 5, 7, 9 and 11 days post infection (dpi), cloacal (CL) and oropharyngeal (OP) swabs were collected from inoculated and contacted animals and titrated in chicken embryos to determine virus shedding using the Reed–Muench method [42]. At 3, 6 and 9 dpi, 3 inoculated chickens and 3 inoculated ducks were sacrificed, and their organs (trachea, lung, heart, liver, kidney, spleen, cecum, duodenum, brain, thymus, pancreas and bursa of Fabricius) were harvested for virus titration. At 3 dpi, lung tissue samples collected were preserved in 10% neutral buffered formalin and then underwent hema-toxylin and eosin (H&E) staining for histopathological examination. At 1 and 3 dpi, the collected lung samples were further used to assess immune-related gene mRNA levels. At 14 and 21 dpi, serum samples from both chickens and ducks were obtained, and antibody levels were determined by the hemagglutination inhibition (HI) test; the HI test was performed as previously described [43]. To test whether the H9N2 virus could transmit from infected chickens to uninfected ducks, eight inoculated chickens were co-housed with five uninfected ducks as a new group, and virus shedding and antibody titers in the contacted ducks were measured following the methods described above.

### 2.4. Assessment of Immune-Related Gene mRNA Levels in the Lungs of Inoculated Chickens and Ducks

Total RNA was obtained from the lung tissues of chickens and ducks at 1 and 3 dpi, and cDNA was synthesized as described in Section 2.1. Primer pairs (Table 1) were selected on the basis of specificity, as determined by dissociation curves. The 20 µL PCR reaction system consisted of 10 µL of 2× SYBR Green qPCR Premix (Yugong Biotech, Lianyungang, China), 0.5 µL of each primer (10 µM), 7 µL of RNase-free water, and 2 µL of cDNA template. Real-time quantitative PCR (RT-qPCR) was performed on an Archimed X4 real-time PCR detection system (ROCGENE, Beijing, China) with the following settings: 95 °C for 30 s, then 40 cycles of 95 °C for 10 s, 60 °C for 10 s, and 72 °C for 30 s. Samples with cycle threshold values < 35 and a single melting peak were considered positive. The relative expression of target genes was determined using the 2^−ΔΔCt^ method and normalized to GAPDH. Each experiment was done three times, and each sample underwent three repetitions.

### 2.5. Mouse Experiment

Female 5-week-old BALB/c mice (Pengyue Experimental Animal Center, Jinan, China) were randomly assigned to two groups: experimental (*n* = 8) and control (*n* = 5). After light anesthesia with CO_2_, experimental mice were dosed intranasally with 50 μL of 10^6^ EID_50_ of the H9N2 virus, whereas control animals were dosed with 50 μL of PBS, as noted earlier [49]. At 3 dpi, organs (lungs, nasal turbinates, kidneys, spleens, and brains) from three euthanized mice were used for virus titration in 9-day-old SPF chicken embryos. The remaining lung tissue samples collected were processed for histopathological analysis, as described in Section 2.3. Meanwhile, the remaining 5 infected mice were observed each day for clinical symptoms, body weight changes, and mortality until 14 dpi.

## 3. Results

### 3.1. The H9N2 AIV Isolated from Chickens in Jiangsu Province, China, 2025

One H9N2 AIV, named A/chicken/Jiangsu/1/2025(H9N2), was isolated from chickens in Xuzhou City, Jiangsu Province, China. The infected chickens were 3-week-old White Leghorns from a commercial flock of around 150,000 birds. Approximately 60% of the flock exhibited clinical signs of infection, with nearly 10,000 deaths recorded. The farm owner confirmed that the flock had never been vaccinated against H9N2 AIV before the outbreak. The diseased chickens showed typical symptoms of influenza infection, including mental depression, loss of appetite, nasal secretion, coughing and sneezing. Necropsy revealed that the lungs of the diseased chickens were enlarged, edematous, blackened, and congested with diffuse pleural thickening and pleural effusion (Figure S1). After PCR amplification, 1% agarose gel electrophoresis was performed to visualize the target fragment, which was consistent with the predicted size. The detailed information for the H9N2 isolate considered in this study is provided in Table S1.

### 3.2. Phylogenetic Analysis of the Chicken-Derived H9N2 AIV

**Figure 1:**
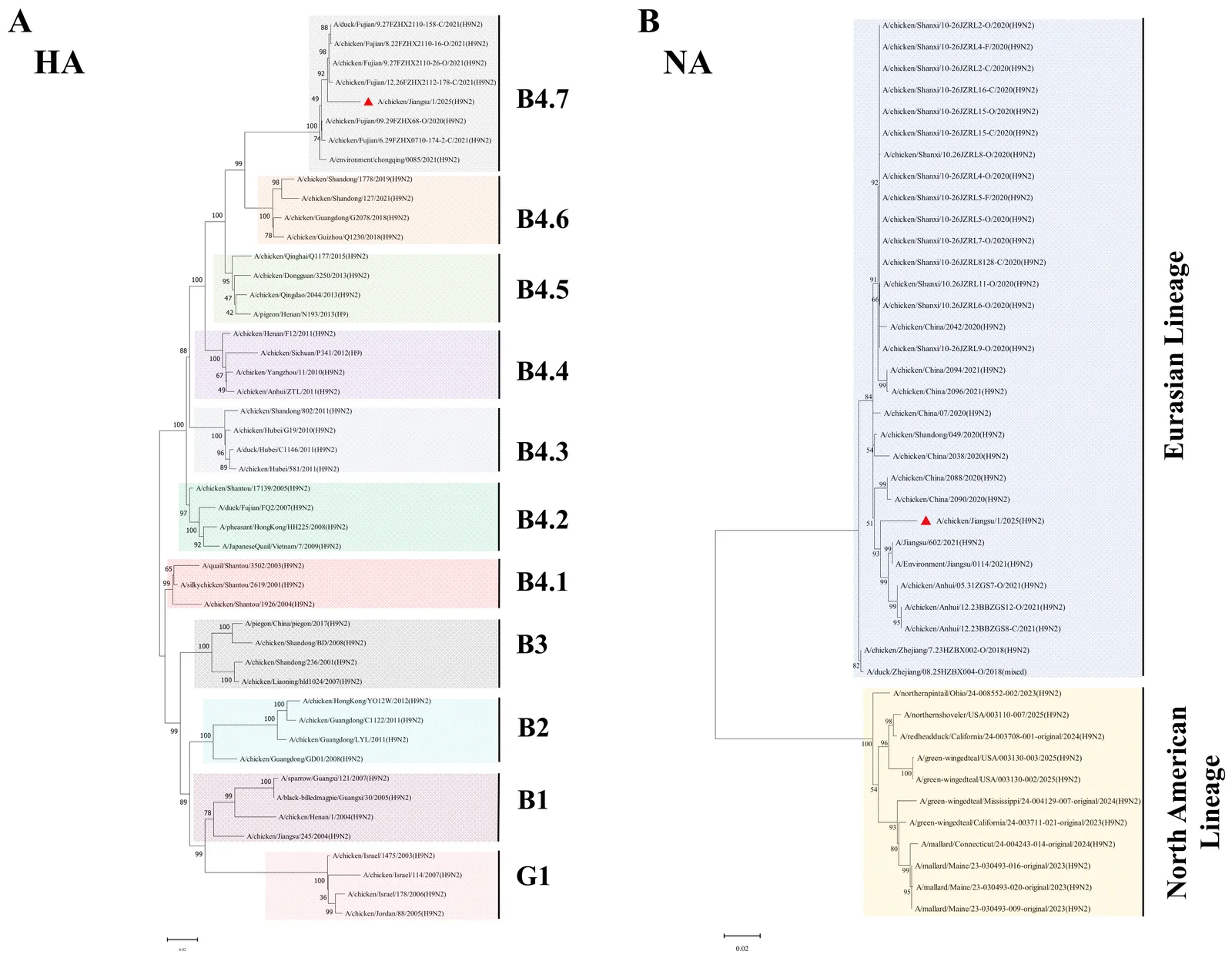
ML phylogenetic trees for the external genes of the chicken-derived H9N2 AIV. HA (**A**) and NA (**B**). Trees were built based on the ML method with 1000 bootstrap replicates. The virus in the present study is indicated by red triangles.

Phylogenetic analysis of the H9N2 isolate was performed in the study; all available reference sequences were acquired from the GISAID EpiFlu database and the NCBI influenza virus database. According to the phylogenetic tree of the external genes, the HA gene (Figure 1A) could be categorized into 11 subclades: G1, B1–B3, and B4.1–B4.7 [39]. Notably, the H9N2 AIV isolate in this study fell into the B4.7 subclade. The NA gene (Figure 1B) was clustered into two major lineages, North American and Eurasian lineages, and A/chicken/Jiangsu/1/2025(H9N2) fell into the Eurasian lineage. Moreover, all internal genes of the H9N2 AIV were clustered in the Eurasian lineage (Figure S2).

### 3.3. The H9N2 AIV from Chickens Carries Molecular Signatures Linked to Enhanced Mammalian Pathogenicity and Polymerase Activity

Molecular analysis demonstrated that the H9N2 AIV in the present study possesses the HA cleavage PSRSSR/GLF motif, suggesting that it is an LPAIV [50]. Notably, a number of amino acid substitutions in this isolate have been identified to enhance mammalian pathogenicity and polymerase activity (Table 2), including I292V and A588V in PB2 [51,52]; D3V and D622G in PB1 protein [53,54]; K356R in PA protein [55]; N30D, I43M and T215A in M1 protein [56,57]; and P42S in NS1 protein [58].

### 3.4. The H9N2 Virus from Chickens Has Undergone Complex Gene Reassortment

**Figure 2:**
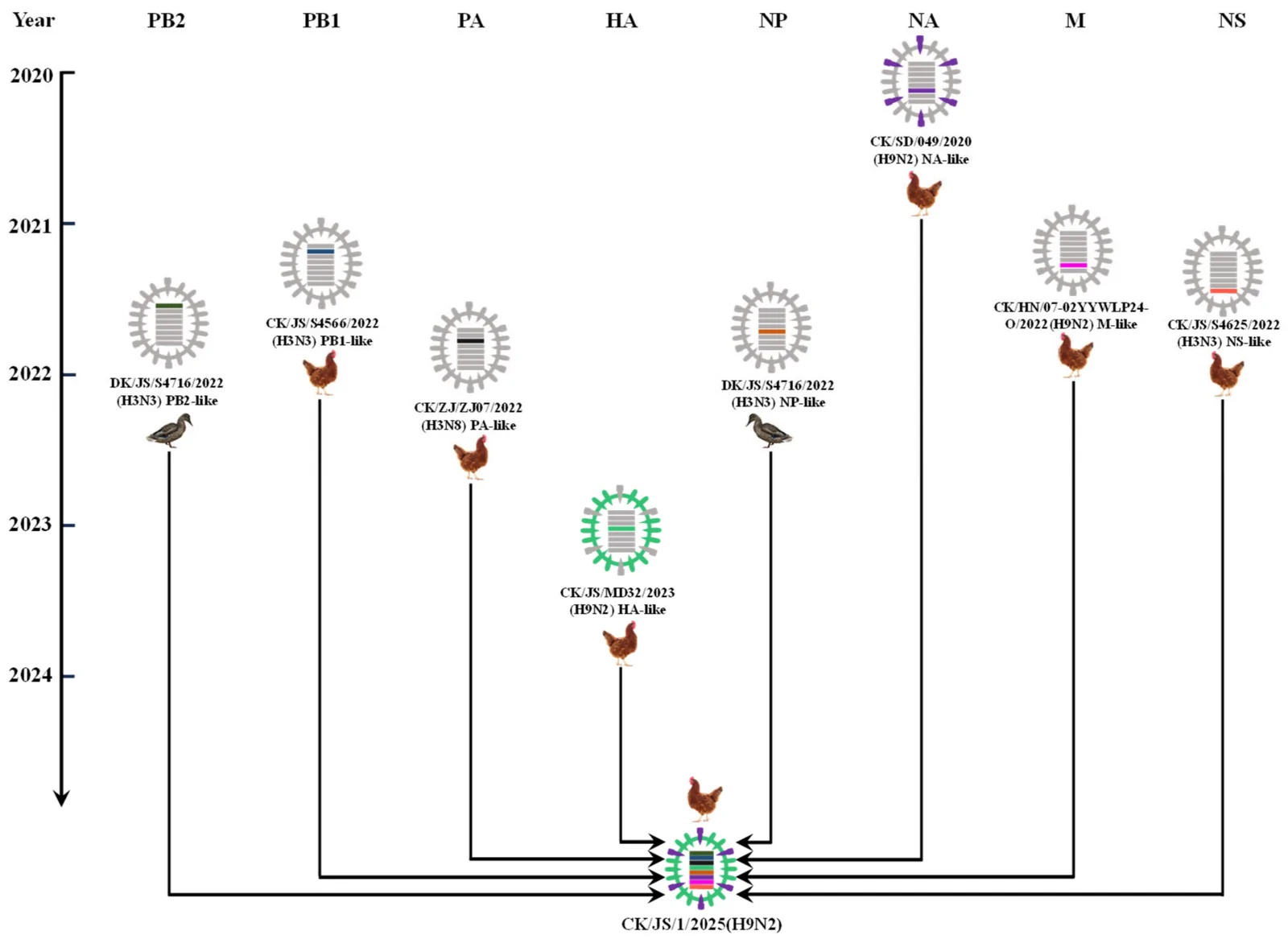
The H9N2 virus gene reassortment schematic. The eight gene fragments in the H9N2 virus (NS, M, NA, NP, HA, PA, PB1, and PB2, from bottom to top) are color-coded, and each color denotes a separate viral genetic background.

To trace the origins of A/chicken/Jiangsu/1/2025(H9N2), we investigated the formation of the virus by blasting the highest homology virus to its eight gene segments from the GISAID EpiFlu database and the NCBI Influenza virus database. As illustrated in Figure 2 and Table S2, the HA, NA and M genes of A/chicken/Jiangsu/1/2025(H9N2) were derived from chicken-origin H9N2 viruses, with CK/JS/MD32/2023(H9N2)-like, CK/SD/049/2020(H9N2)-like and CK/HN/07-02YYWLP24-O/2022(H9N2)-like acting as their gene donors, respectively. The PA gene originated from a chicken-derived H3N8 virus (CK/ZJ/ZJ07/2022(H3N8)-like). Additionally, the PB1 and NS genes were sourced from chicken-derived H3N3 viruses, with CK/JS/S4566/2022(H3N3)-like and CK/JS/S4625/2022(H3N3)-like being their gene donors, respectively. Lastly, both PB2 and NP genes were obtained from the same duck-derived H3N3 isolate (DK/JS/S4716/2022(H3N3)-like). These data indicated that the emergence of A/chicken/Jiangsu/1/2025(H9N2) resulted from complex genetic recombination and that this virus was derived from various LPAIV subtypes in poultry.

### 3.5. Effective Replication and Transmissibility of the Chicken-Derived H9N2 AIV in Chickens

**Figure 3:**
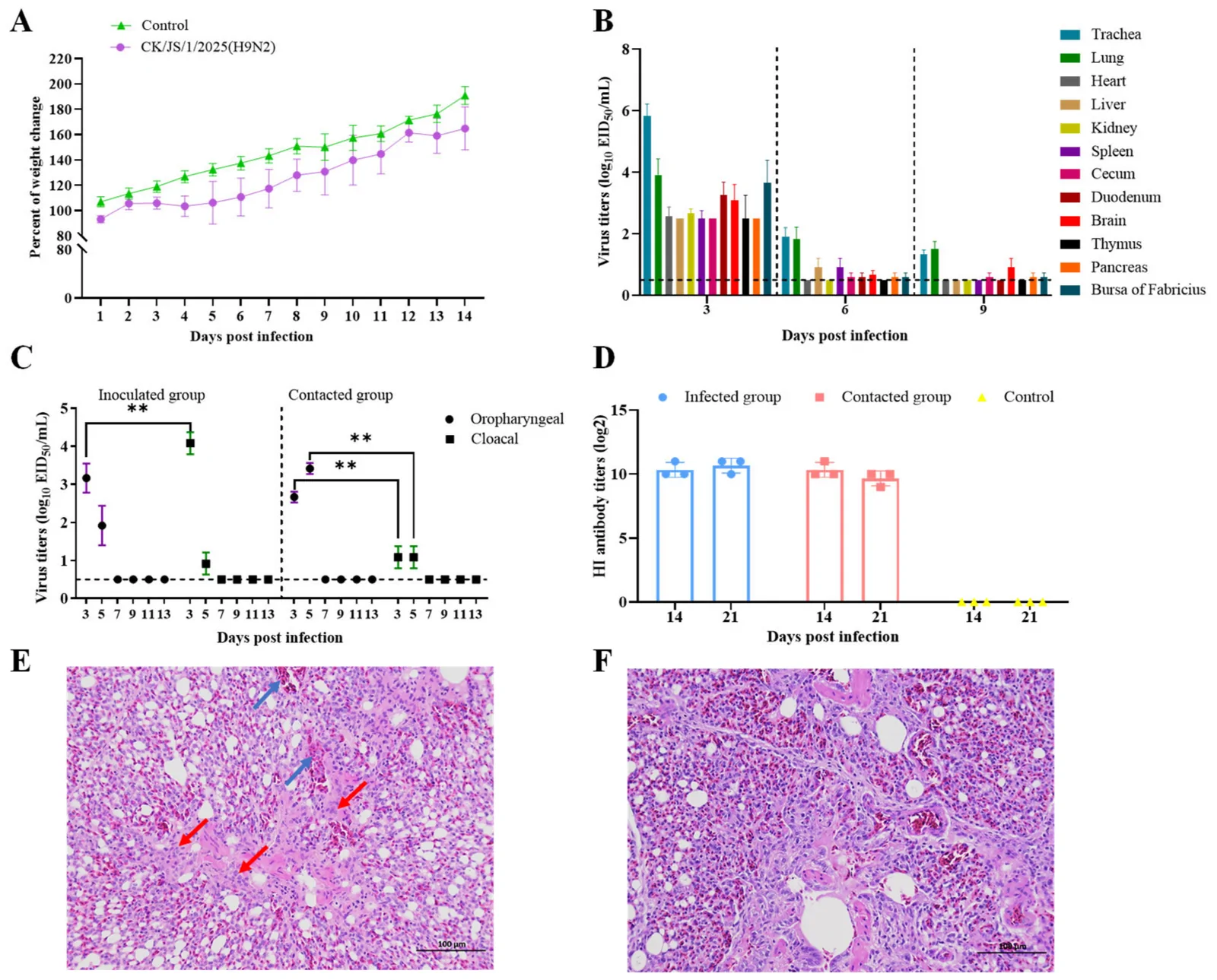
Pathogenicity, transmissibility, and serological response to H9N2 virus in SPF chickens. (**A**) Changes in body weight in the inoculated and control groups (*n* = 5 per group) after inoculation with 10^6^ EID_50_ of the H9N2 virus. (**B**) Viral titers in all tested organs of SPF chickens at 3, 6 and 9 dpi. (**C**) Virus titers were tested in OP and CL swabs from inoculated and contacted chickens. The minimum detection threshold for the H9N2 virus is indicated by the dashed line, and the error bars represent the standard deviations. The assessment of statistical significance was conducted using the Student’s *t*-test (** *p* < 0.01). (**D**) HI antibody titers were tested in inoculated and contacted chickens at 14 and 21 dpi. (**E**) Representative H&E image of lungs from chickens infected with H9N2 virus at 3 dpi. Vascular stasis (blue arrows), inflammatory cell infiltration (red arrows). (**F**) Representative H&E image of lungs from control chickens at 3 dpi. Scale bar represents 200×, 100 μm.

To investigate the pathogenicity and transmissibility of the chicken-derived H9N2 AIV, 3-week-old SPF chickens were inoculated with 100 μL of 10^6^ EID_50_ of A/chicken/Jiangsu/1/2025(H9N2) via the intranasal route. As shown in Figure 3A, chickens inoculated with the H9N2 virus exhibited a transient loss of body weight at 1 dpi and gradually recovered at 2 dpi. However, body weights in the inoculated group remained markedly lower after inoculation than those in the controls. In addition, the virus was found in all examined organs of the inoculated chickens, with the viral titer peaking at 3 dpi and gradually declining at 6 and 9 dpi (Figure 3B). Furthermore, at 3 and 5 dpi, the H9N2 virus was found in the OP and CL swabs from both inoculated and contact chickens (Figure 3C, Table S3). During the observation period, there were no clinical signs in any of the chickens, and all inoculated and contacted chickens showed seroconversion at 14 and 21 dpi (Figure 3D, Table S3). Histopathological examination revealed vascular congestion and mild interstitial infiltration of inflammatory cells in the lungs of inoculated chickens; conversely, no marked pathological change was found in the control group (Figure 3E,F). Overall, the H9N2 virus could replicate and transmit in chickens and induce high HI antibody titers.

### 3.6. Poor Replication and Transmissibility of the Chicken-Derived H9N2 AIV in Ducks

**Figure 4:**
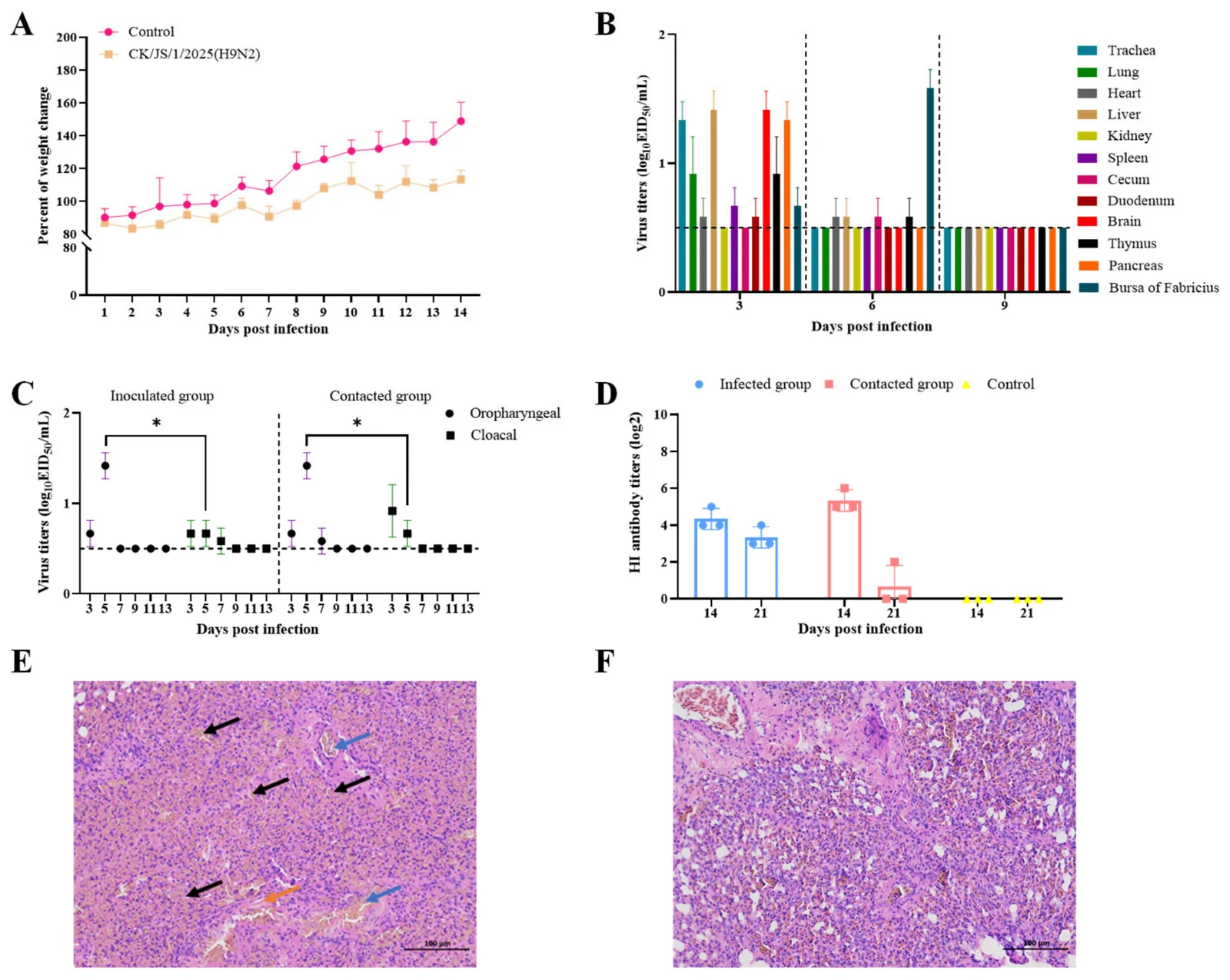
Pathogenicity, transmissibility, and serological response to H9N2 virus in SPF ducks. (**A**) Changes in body weight in the inoculated and control groups (*n* = 5 per group) after inoculation with 10^6^ EID_50_ of the H9N2 virus. (**B**) Viral titers in all tested organs of SPF ducks were tested at 3, 6 and 9 dpi. (**C**) Virus titers were tested in OP and CL swabs from inoculated and contacted ducks. The minimum detection threshold for the H9N2 virus is indicated by the dashed line, and the error bars represent the standard deviations. The assessment of statistical significance was conducted using the Student’s *t*-test (* *p* < 0.05). (**D**) HI antibody titers were tested in inoculated and contacted duck at 14 and 21 dpi. (**E**) Representative H&E image of lungs from ducks infected with H9N2 virus at 3 dpi. Alveolar hemorrhage (black arrows), vascular stasis (blue arrows) and slight fibrin exudation (orange arrow). (**F**) Representative H&E image of lungs from control ducks at 3 dpi. Scale bar represents 200×, 100 μm.

To investigate the pathogenicity and transmissibility of the H9N2 virus in SPF ducks, 3-week-old SPF ducks were inoculated with 100 μL of 10^6^ EID_50_ of A/chicken/Jiangsu/1/2025(H9N2) via the intranasal route. As illustrated in Figure 4A, ducks infected with the H9N2 virus suffered slight weight loss at 1 dpi, while they started to recover gradually at 9 dpi; similar to the findings in chickens, body weights in the inoculated group were also markedly lower than in the controls. Furthermore, the virus replicated poorly in ducks, with low viral titers detected in multiple organs at 3 and 6 dpi, whereas the virus was not found at 9 dpi (Figure 4B). In addition, at 3 and 5 dpi, the virus was found in both OP and CL swabs from inoculated and contacted ducks, whereas it was found only in CL swabs of inoculated ducks and OP swabs of contacted ducks at 7 dpi (Figure 4C, Table S4). Throughout the observation period, all tested ducks survived without any clinical symptoms, and seroconversion occurred in inoculated ducks at 14 and 21 dpi, while in contacted ducks it occurred only at 14 dpi (Figure 4D, Table S4). Histopathological examination revealed massive hemorrhage and stasis in the alveoli, intravascular stasis, and a small amount of fibrinous exudate of the lungs from inoculated ducks, whereas the control group exhibited no significant pathological changes (Figure 4E,F). In general, the viral replication titers in chickens were higher than those in ducks (Figure S3). However, despite the low viral titers observed in ducks, the virus still retained the capacity for horizontal transmission.

### 3.7. The H9N2 Virus from Chickens Could Be Transmitted to Contacted Ducks Through Infected Chickens

**Figure 5:**
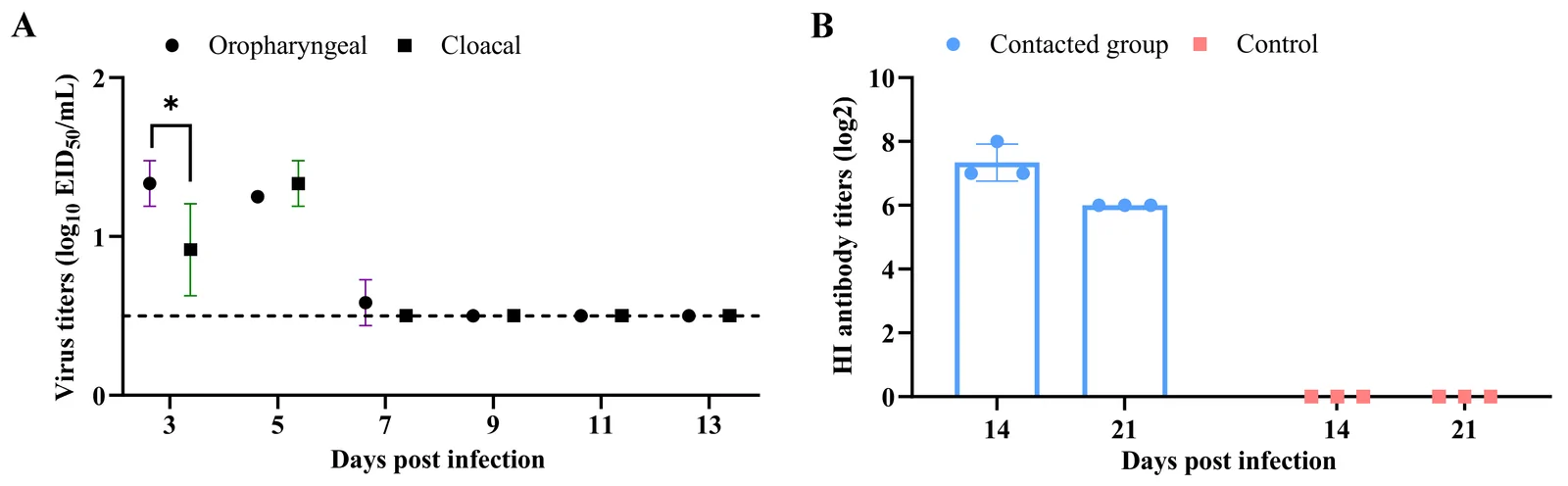
Transmissibility and serological response to H9N2 virus in contacted ducks through infected chickens. (**A**) Virus titers were tested in OP and CL swabs from contacted ducks. (**B**) HI antibody titers of contacted ducks at 14 and 21 dpi. The minimum detection threshold for the H9N2 virus is indicated by the dashed line, and the error bars represent the standard deviations. The assessment of statistical significance was conducted using the Student’s *t*-test (* *p* < 0.05).

To investigate whether the H9N2 virus could be transmitted from chickens to ducks, five ducks were housed with chickens inoculated with A/chicken/Jiangsu/1/2025(H9N2) as a new contacted group. The results indicated that viral shedding was found in CL and OP swabs from all contacted ducks at 3 and 5 dpi and was detected only in OP swabs at 7 dpi (Figure 5A, Table S5). Furthermore, we observed that all contacted ducks showed seroconversion at 14 and 21 dpi (Figure 5B, Table S5). These results indicate that the A/chicken/Jiangsu/1/2025(H9N2) virus could transmit from chickens to ducks with higher transmission capability than that from ducks to ducks.

### 3.8. Chickens and Ducks Exhibit Different Immune Responses to H9N2 AIV Infection

**Figure 6:**
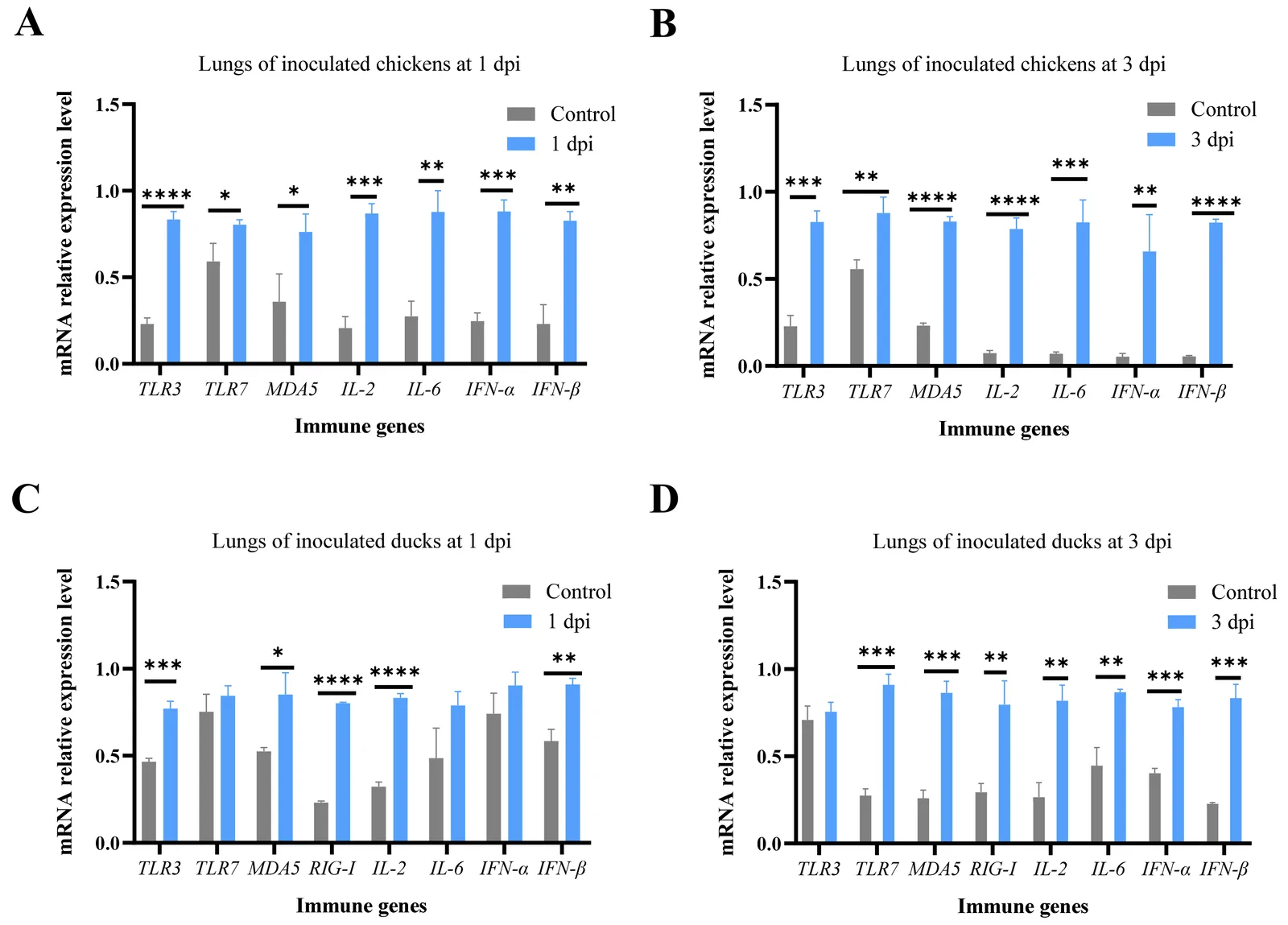
mRNA expression levels of immune-related genes in the lungs of inoculated chickens and ducks. Relative mRNA expression of TLR3, TLR7, MDA5, IL-2, IL-6, IFN-α and IFN-β in the lungs of chickens inoculated with the H9N2 AIV at (**A**) 1 dpi and (**B**) 3 dpi. Relative mRNA expression of TLR3, TLR7, MDA5, RIG-I, IL-2, IL-6, IFN-α and IFN-β in the lungs of ducks inoculated with the H9N2 AIV at (**C**) 1 dpi and (**D**) 3 dpi. Each bar indicates the mRNA level of each target gene, normalized to GAPDH. The assessment of statistical significance was conducted using the Student’s *t*-test (* *p* < 0.05; ** *p* < 0.01; *** *p* < 0.001; **** *p* < 0.0001).

To assess the immune reactions of chickens and ducks inoculated with the H9N2 virus, we used RT-qPCR to measure the relative mRNA levels of immune genes at 1 and 3 dpi. In the lungs of inoculated chickens, the mRNA levels of TLR3, TLR7, MDA5, IL-2, IL-6, IFN-α and IFN-β were increased at 1 dpi and were significantly upregulated at 3 dpi (Figure 6A,B). In the lungs of inoculated ducks, at 1 dpi, mRNA levels of TLR7, IL-6, and IFN-α showed no significant increase, while those of TLR3, MDA5, RIG-I, IL-2, and IFN-β were significantly elevated. At 3 dpi, except for the mRNA levels of TLR3, the mRNA levels of all other tested immune genes were significantly elevated (Figure 6C,D). Collectively, in comparison with controls, the mRNA expression levels of all tested genes were upregulated in the lungs of both chickens and ducks at 1 and 3 dpi. Moreover, with the exception of TLR7 and MDA5, the other immune genes in inoculated ducks were overall lower than those in chickens.

### 3.9. The H9N2 Virus from Chickens Could Infect Mice Without Requiring Prior Adaptation

**Figure 7:**
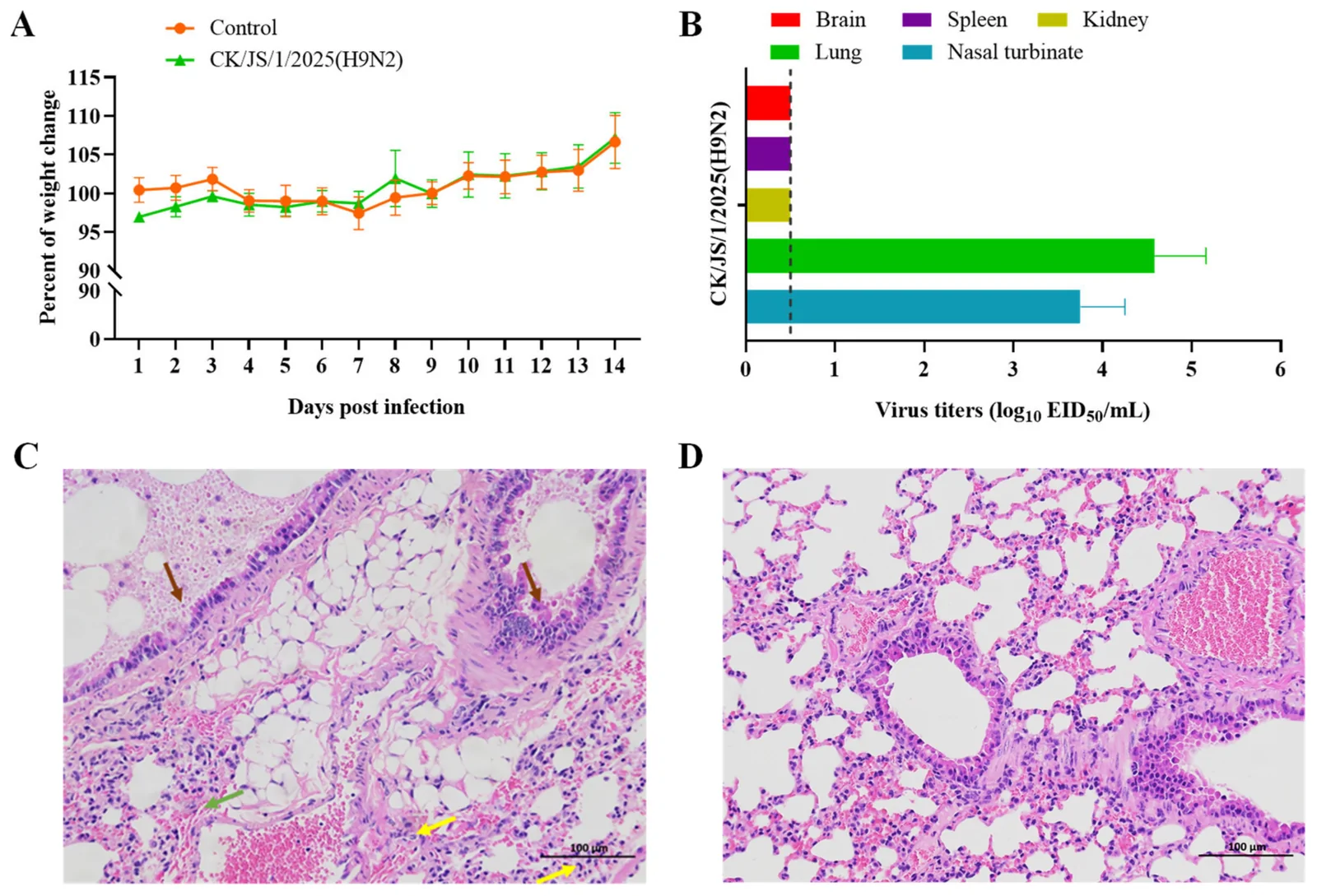
Pathogenicity and replication of H9N2 virus in mice. (**A**) Changes in body weight in the inoculated and control groups (*n* = 5 per group) after inoculation with 10^6^ EID_50_ of the H9N2 virus. (**B**) Virus titers were assessed in all test organs of mice at 3 dpi. The minimum detection threshold for the H9N2 virus is indicated by the dashed line, and the error bars represent the standard deviations. (**C**) Histopathological changes were found in the mouse lungs at 3 dpi. Occasional granulocyte infiltration was observed in the alveolar walls (yellow arrows), with mild focal thickening of the alveolar walls and expansion of the interalveolar septa. Mild perivascular hemorrhage was noted (green arrow), along with desquamation of the bronchiolar epithelium (brown arrows). (**D**) Histopathological sections of control group mice. Scale bar represents 200×, 100 μm.

To evaluate the virulence and replicability of A/chicken/Jiangsu/1/2025(H9N2) in mice, BALB/c mice were inoculated with 50 μL of 10^6^ EID_50_ of the H9N2 AIV. As illustrated in Figure 7A, the mice inoculated with the H9N2 virus showed slight weight loss at 1 dpi, while their weight began to recover gradually at 8 dpi. Furthermore, at 3 dpi, we detected A/chicken/Jiangsu/1/2025(H9N2) in the lungs and nasal turbinates of all three inoculated mice, with titers in the lungs ranging from 4.25 to 5.25 log_10_ EID_50_ and those in the nasal turbinates ranging from 3.25 to 4.25 log_10_ EID_50_ (Figure 7B). In contrast, the virus was not found in any of the other tested organs (kidney, spleen, or brain) of the infected mice. Histopathological examination revealed thickened alveolar walls, widened alveolar septa, mild perivascular hemorrhage, desquamation of bronchiolar epithelium, and occasional granulocyte infiltration in the alveolar walls of the lungs from inoculated mice, whereas the control group exhibited no obvious pathological change (Figure 7C,D). Collectively, A/chicken/Jiangsu/1/2025(H9N2) did not cause mortality in mice, whereas it was able to infect BALB/c mice without prior adaptation, replicate efficiently in the nasal turbinates and lungs, and induce notable pulmonary lesions.

## 4. Discussion

Since its first isolation in turkeys in 1966, H9N2 AIV has spread rapidly and has emerged as the most widespread AIV subtype in poultry, overtaking the previously dominant H5N6 and H7N9 AIVs [36]. The H9N2 AIVs normally caused a decrease in egg production and respiratory signs and impaired immune function, and infected chickens were extremely susceptible to mixed infections with infectious bronchitis virus, laryngotracheitis virus, and *Escherichia coli*, which caused severe economic damage to poultry production [59,60,61,62]. In March 2025, one chicken-origin H9N2 AIV, named A/chicken/Jiangsu/1/2025(H9N2), was isolated in Xuzhou City, Jiangsu Province, China. Phylogenetic analysis indicated that the HA gene fragment of the H9N2 AIV fell into the B4.7 subclade within the Eurasian lineage, and the remaining seven gene fragments were also classified within the Eurasian lineage. However, given that all the results originate from one single isolate, they may not represent the overall genetic diversity or pathogenicity of H9N2 AIV. Therefore, broader surveillance and larger sample collections are required to systematically evaluate these traits.

Gene reassortment is a pivotal driver of the evolution of AIV and the emergence of pandemic isolates [63].It has been documented that the chicken-derived H9N2 AIV contributed all its internal gene segments to the new human-infecting H7N9, H5N6, and H10N8 AIVs that emerged in China [64,65,66,67]. In this study, the emergence of A/chicken/Jiangsu/1/2025(H9N2) was attributed to complex genetic reassortment among different LPAIV subtypes in China. Nevertheless, it is important to point out that the reassortment analysis is based on BLAST homology searches rather than proven gene donor identification, and this limitation should be taken into consideration. Consequently, the continued reassortment of H9N2 AIVs requires close public attention.

IAVs are prone to adaptive mutations during their evolution to enhance their replication efficiency in mammals [68]. In this study, the A/chicken/Jiangsu/1/2025(H9N2) isolate harbored amino acid substitutions, including I292V and A588V in PB2 protein [51,52]; D3V and D622G in PB1 protein [53,54]; K356R in PA protein [55]; N30D, I43M and T215A in M1 protein [56,57]; and P42S in NS1 protein [58], which may enhance the viral mammalian pathogenicity and polymerase activity. However, these inferences are based solely on literature data and have not been experimentally validated in the present study. Thus, further functional investigations are required to confirm these effects. Moreover, given that the phenotypic effects of these mutations are primarily derived from studies on other AIV subtypes, cross-subtype extrapolation of mutational effects should be interpreted with caution. In this regard, whether these mutations are a common feature of the B4.7 lineage H9N2 AIV requires verification through broader sequence comparisons and temporal dynamic analysis, which will be a focus of our future research. To evaluate the capacity of A/chicken/Jiangsu/1/2025(H9N2) to infect mammals, in vivo experiments were conducted. The results demonstrated that the chicken-derived H9N2 isolate could infect BALB/c mice without requiring prior adaptation and that it replicated effectively in the nasal turbinates and lungs of mice. We acknowledge that the sample size of our mouse experiment is relatively modest; however, it is consistent with common practice in similar studies [49,69,70], and the statistical significance of the observed differences supports the validity of our conclusions. Interestingly, the dissociation between the mild clinical signs and the detectable viral titers in mice cannot be fully explained by our current data, and this phenomenon warrants further investigation. These results demonstrate that the potential risks posed by chicken-derived H9N2 viruses should not be overlooked, and continuous monitoring of H9N2 viruses should be enhanced.

H9N2 AIV is widely circulating in poultry, posing a threat to the poultry industry and public health [71]. In this research, we evaluated the pathogenicity and transmissibility of A/chicken/Jiangsu/1/2025(H9N2) in SPF chickens and ducks. The findings demonstrated that the virus could replicate and transmit more efficiently in chickens than in ducks. Some researchers have shown that poultry and waterfowl exhibit a differential response to AIVs, as both local mucosal and innate immunity may play a key role in host resistance to influenza [72,73]. We acknowledge that our sample sizes were modest; nevertheless, they were consistent with common practice in similar studies [74,75]. More importantly, virus transmission experiments showed that the H9N2 isolate from chickens could be transmitted to contacted ducks through infected chickens and induce high HI antibody production in contacted ducks. These findings demonstrate that the H9N2 virus is capable of cross-species transmission between chickens and ducks. This cross-species transmission may facilitate sustained viral circulation and create critical opportunities for adaptive evolution within hosts with different genetic backgrounds, resembling the co-housing of multiple avian species in live poultry markets, thereby highlighting its real-world epidemiological relevance [20].

The innate immune system serves as the primary line of defense against AIV infection and recognizes pathogen-associated molecular patterns (PAMPs) through PRRs, which trigger antiviral signaling cascades and induce the production of IFNs, cytokines, and chemokines [76]. This study compared the innate immune responses in the lungs of chickens and ducks infected with the H9N2 virus. The findings revealed that the mRNA expression levels of key immune genes were upregulated in the lungs of both chickens and ducks at 1 and 3 dpi; however, the overall fold increase in ducks was lower than that in chickens. The higher viral loads in chickens than in ducks after infection suggested that an excessive inflammatory response to viral infection may recruit inflammatory cells to infected tissues, thus promoting viral spread [77]. However, it remains unclear whether this association reflects a direct causal link, as the relationship could be bidirectional or both phenomena might arise from intrinsic differences in host tropism between chickens and ducks.

H9N2 viruses are prone to antigenic variation under immune pressure [75]. Since our study did not include challenge–protection experiments, we cannot assess the vaccine-escape potential of our isolate. However, it has been experimentally demonstrated that the candidate vaccine strain R118-A reduced shedding of the homologous B4.7.4 lineage H9N2 AIV and conferred cross-protection against the heterologous B4.7.2 lineage H9N2 AIV [78]. Accordingly, challenge–protection studies are required to clarify antigenic matching profiles of our isolate against current vaccine strains and evaluate the escape risk in future research.

## 5. Conclusions

In summary, one newly isolated H9N2 AIV belongs to the B4.7 subclade within the Eurasian lineage and is a reassortant originating from multiple AIV subtypes circulating in China. Our findings revealed that the H9N2 AIV considered in the present study had acquired several mammalian-adaptive mutations, replicated effectively in BALB/c mice without prior adaptation, and was able to replicate and transmit in chickens and ducks. Notably, the virus was also capable of transmission from chickens to ducks. The mRNA expression of immune-related genes was upregulated in the lungs of chickens and ducks inoculated with the H9N2 AIV at 1 and 3 dpi. These results provided insight into the evolution, pathogenic characteristics, and host antiviral immune responses of the chicken-origin H9N2 AIVs.

## Figures and Tables

**Table 1:** RT-qPCR primer sequences.

Species	Genes	Forward Primers (5′–3′)	Reverse Primers (5′–3′)	Cited
Duck	TLR3	GAGTTTCACACAGGATGTTTAC	GTGAGATTTGTTCCTTGCAG	[44]
	TLR7	CCTTTCCCAGAGAGCATTCA	TCAAGAAATATCAAGATAATCACATCA	[45]
	MDA5	CTTGCAGATGATTTAAGTGGA	CTTCACTACAGAATGTCCTGG	[45]
	RIG-I	CTGGCAGAAGCAATTGAGAAC	TGCTGAATCTCTTCACACTCC	[45]
	IL-2	GCCAAGAGCTGACCAACTTC	ATCGCCCACACTAAGAGCAT	[46]
	IL-6	TTCGACGAGGAGAAATGCTT	CCTTATCGTCGTTGCCAGAT	[44]
	IFN-α	TCCTCCAACACCTCTTCGAC	GGGCTGTAGGTGTGGTTCTG	[44]
	IFN-β	AGATGGCTCCCAGCTCTACA	AGTGGTTGAGCTGGTTGAGG	[44]
	GAPDH	ATGTTCGTGATGGGTGTGAA	CTGTCTTCGTGTGTGGCTGT	[44]
Chicken	TLR3	CTCTATTCCTTGCTGGAACA	CTCGGTCAGATTTTCAGGATCT	[47]
	TLR7	CGTCTGCTGAGTCTGGAAGC	ACGTTGAAGATAAGTTGGGTGG	[47]
	MDA5	GGACGACCACGATCTCTGTGT	CACCTGTCTGGTCTGCATGTTATC	[48]
	IL-2	CGGGATCCATGATGTGCAAAGTACTG	CGGTCGACTTATTTTTGCAGATATCT	[46]
	IL-6	ATGTGCAAGAAGTTCACCGTG	TTCCAGGTAGGTCTGAAAGGCGAA	[46]
	IFN-α	ATGCCACCTTCTCTCACGAC	AGGCGCTGTAATCGTTGTCT	[46]
	IFN-β	CCTCAACCAGATCCAGCATT	GGATGAGGCTGTGAGAGGAG	[46]
	GAPDH	CCTCTCTGGCAAAGTCCAAG	CATCTGCCCATTTGATGTTG	[46]

**Table 2:** Molecular signatures of the H9N2 AIV A/chicken/Jiangsu/1/2025(H9N2) considered in the study.

Protein	Amino Acid Position/Motif	Phenotypic Consequences	CK/JS/1/2025(H9N2)
HA (H3 numbering) ^a^	T190V	Enhanced replication in mammalian cells	-
	Q226L	Enhanced virus binding to human-type receptor	-
	G228S	Enhanced virus binding to human-type receptor	- ^b^
NA (N2 numbering) ^a^	Stalk deletion	Enhanced virulence in mice	-
	E119G	Decreased susceptibility to zanamivir	-
	N294S	Decreased susceptibility to oseltamivir	-
PB2	D253N	Enhanced polymerase activity	-
	I292V	Enhanced virulence in mice and increased polymerase activity	V
	A588V	Enhanced virulence in mice and increased polymerase activity	V
	Q591K	Enhanced virulence in mice and increased polymerase activity	-
	E627K	Enhanced virulence in mice and increased polymerase activity	-
	D701N	Enhanced virulence in mice and increased polymerase activity	-
	S714R	Enhanced virulence in mice and increased polymerase activity	-
PB1	D3V	Enhanced virulence in mice and increased polymerase activity	V
	K577E	Enhanced virulence in mice and increased polymerase activity	-
	D622G	Enhanced virulence in mice and increased polymerase activity	G
PA	K356R	Enhanced virulence in mice and increased polymerase activity	R
	N383D	Enhanced polymerase activity	-
NP	E434K	Enhanced polymerase activity	-
M1	N30D	Increased virulence in mice	D
	I43M	Enhanced virulence in mice	M
	T215A	Enhanced virulence in mice	A
NS1	80–84 deletion	Increased virulence in mice	-
	P42S	Increased virulence in mice	S

^a^: The numbering of mutations and motifs follows the alignments with A/Aichi/2/1968(H3N2). ^b^: The “-” denotes that the mutation was not found in the H9N2 virus.

## Data Availability

Genome sequences of the H9N2 AIV in this study were uploaded to the GISAID EpiFlu database (accession ID: EPI5102519–EPI5102526).

## Supplementary Materials

The following supporting information can be downloaded at https://www.mdpi.com/article/10.3390/microorganisms14081667/s1, Figure S1: Postmortem examination of lung from chickens infected with H9N2 virus. Figure S2: ML phylogenetic trees for the internal genes of the H9N2 isolate from chickens: (A) PB2 gene, (B) PB1 gene, (C) PA gene, (D) NP gene, (E) M gene and (F) NS gene. Virus in the present study is indicated by red triangles. Figure S3: Comparison of viral titers in various organs between chickens and ducks at 3 dpi (A), 6 dpi (B) and 9 dpi (C). Table S1: H9N2 virus isolated from chickens in Jiangsu Province, China, 2025. Table S2: The highest nucleotide homology virus of the entire genomes of the A/chicken/Jiangsu/1/2025(H9N2). Table S3: Viral shedding and antibody titers in SPF chickens inoculated with the H9N2 virus. Table S4: Virus shedding and antibody titers of SPF chickens inoculated with H9N2 virus. Table S5: Virus shedding and antibody titers of contacted ducks through chickens inoculated with H9N2 virus.

## Abbreviations

The following abbreviations are used in this manuscript:

AIV	Avian influenza virus
PRRs	Pattern-recognition receptors
HA	Hemagglutinin
NA	Neuraminidase
IAVs	Influenza A viruses
HPAIV	Highly pathogenic avian influenza virus
LPAIV	Low pathogenic avian influenza virus
IVPI	Intravenous pathogenicity index
SDAU	Shandong Agricultural University
SPF	Specific-pathogen-free
EID_50_	50% embryo infective dose
p.i.	Post-infection
PBS	Phosphate-buffered saline
H&E	Hematoxylin and eosin
OP	Oropharyngeal
CL	Cloacal
HI	Hemagglutination inhibition
ML	Maximum likelihood
RT-qPCR	Real-time quantitative PCR
PAMPs	Pathogen-associated molecular patterns
