# Supplementary material for: Genetic and Pathogenicity Studies of Avian Influenza (H9N2) Virus Isolated from Chickens in Jiangsu Province, China, in 2025
Source: Microorganisms. 2026 Jul 30;14(8):1667. doi: 10.3390/microorganisms14081667 (PMC13515321; doi:10.3390/microorganisms14081667)
Supplement: Supplementary file 1 [file microorganisms-14-01667-s001.zip › microorganisms-4415479-supplementary.pdf]

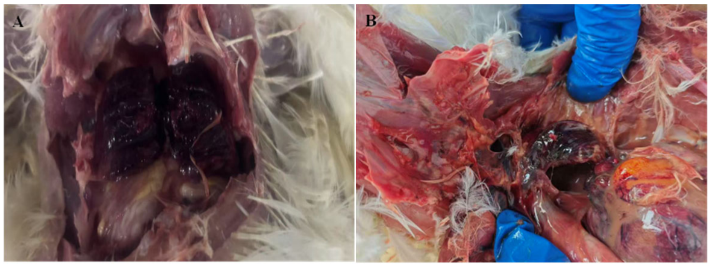

Figure S1. Postmortem examination of lung from chickens infected with H9N2 virus.

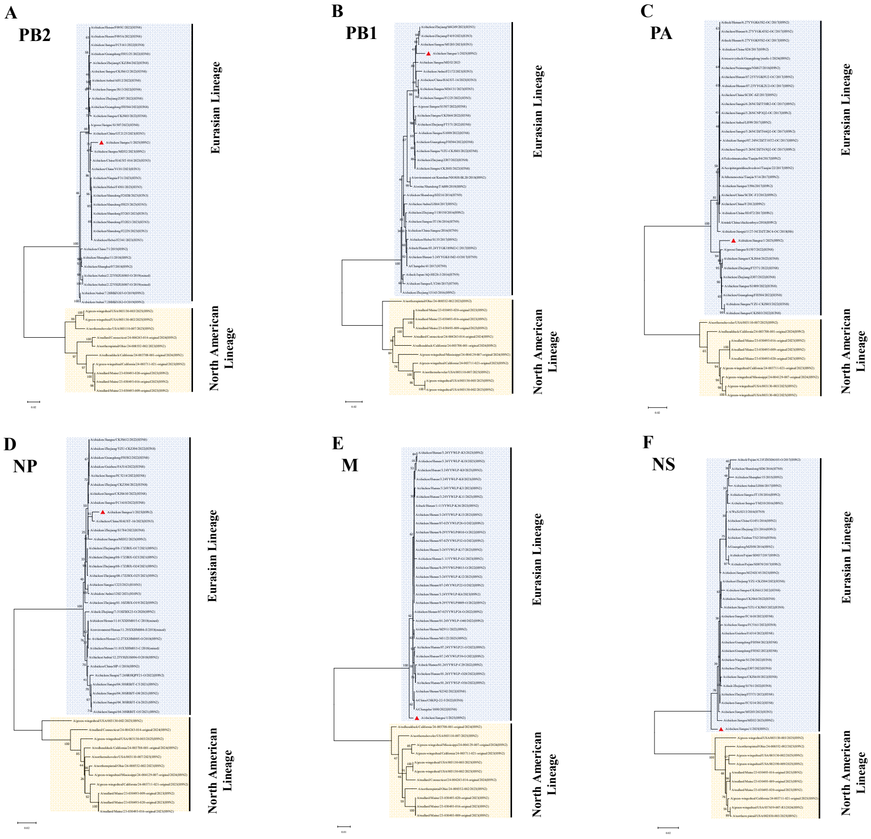

Figure S2. ML phylogenetic trees for the internal genes of the H9N2 isolate from chickens. (A) PB2 gene, (B) PB1 gene, (C) PA gene, (D) NP gene, (E) M gene and (F) NS gene. Virus in the present study is indicated by red triangles

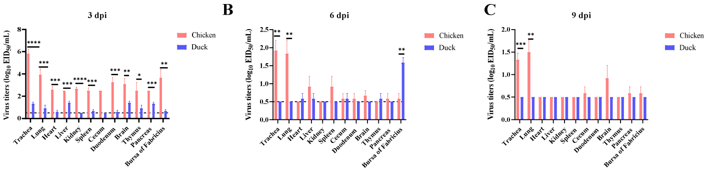

Figure S3. Comparison of viral titers in various organs between chickens and ducks at 3 dpi (A),6 dpi (B) and 9 dpi (C).

Table S1. H9N2 virus isolated from chickens in Jiangsu Province, China, 2025

Sample		AIV isolate			H9N2 AIV	
No.	Type	No.	Positive rate (%)	Subtype (No.)	Name	Abbreviation
326	Tissue Swab	1	0.3	H9N2	A/chicken/Jiangsu/1/2025	CK/JS/1/2025

Table S2. The highest nucleotide homology virus of the entire genomes of the A/chicken/Jiangsu/1/2025(H9N2)

Segment	Highest homologous strain			
	**Name**	**Abbreviation**	**Homology**	**Accession**
PB2	A/duck/Jiangsu/S4716/2022(H3N3)	DK/JS/S4716/2022(H3N3)	98.86%	EPI4314307
PB1	A/chicken/Jiangsu/S4566/2022(H3N3)	CK/JS/S4566/2022(H3N3)	98.77%	EPI4314276
PA	A/chicken/Zhejiang/ZJ07/2022(H3N8)	CK/ZJ/ZJ07/2022(H3N8)	98.7%	PQ676061.1
HA	A/chicken/Jiangsu/MD32/2023(H9N2)	CK/JS/MD32/2023(H9N2)	98.57%	EPI4307500
NP	A/duck/Jiangsu/S4716/2022(H3N3)	DK/JS/S4716/2022(H3N3)	99.26%	EPI4314303
NA	A/chicken/Shandong/049/2020(H9N2)	CN/SD/049/2020(H9N2)	97.36%	EPI2200627
M	A/chicken/Hunan/07-02YYWLP24-O/2022(H9N2)	CK/HN/07.02YYWLP24-O/2022(H9N2)	99.39%	EPI4174781
NS	A/chicken/Jiangsu/S4625/2022(H3N3)	CK/JS/S4625/2022(H3N3)	99.80%	EPI4314288

Table S3. Viral shedding and antibody titers in SPF chickens inoculated with the H9N2 virus

Virus	Route	Virus titer ($\log_{10}$ EID$_{50}$[1]/0.1 mL, mean ± SD)						Seroconversion (log2)	
		**3 dpi**[2]	**5 dpi**	**7 dpi**	**9 dpi**	**11 dpi**	**13 dpi**	**14 dpi**	**21 dpi**
CK/JS/1/2025(	Inoculated	3.17±0.	1.91±0.	ND[5]	ND	ND	ND	10-11	10-11

H9N2)	OP[3]	38[4] (3/3)[6]	52 (3/3)	(0/3)	(0/3)	(0/3)	(0/3)	(3/3)	(3/3)
	Inoculated CL[7]	4.08±0.29 (3/3)	0.92±0.29 (3/3)	ND (0/3)	ND (0/3)	ND (0/3)	ND (0/3)		
	Contacted OP	2.67±0.14 (3/3)	3.42±0.14 (3/3)	ND (0/3)	ND (0/3)	ND (0/3)	ND (0/3)	10-11 (3/3)	9-10 (3/3)
	Contacted CL	1.08±0.29 (3/3)	1.08±0.29 (3/3)	ND (0/3)	ND (0/3)	ND (0/3)	ND (0/3)		

[1] EID_{50}, 50% embryo infective dose;

[2] DPI, days post infection;

[3] OP, oropharyngeal swab sample;

[4] The viral titers are shown as the mean ± SD from three separate experiments;

[5] ND, not detected;

[6] Positive birds / total birds sampled per time point;

[7] CL, cloacal swab sample.

Table S4. Virus shedding and antibody titers of SPF ducks inoculated with H9N2 virus

**Virus**	**Route**	**Virus titer (log_{10} EID_{50}[1]/0.1 mL, mean ± SD)**						**Seroconversion (log2)**	
		**3 dpi[2]**	**5 dpi**	**7 dpi**	**9 dpi**	**11 dpi**	**13 dpi**	**14 dpi**	**21 dpi**

CK/JS/1/2025(H9N2)	Inoculated OP[3]	0.67±0.14[4] (2/3)[6]	1.42±0.14 (3/3)	ND[5] (0/3)	ND (0/3)	ND (0/3)	ND (0/3)	4-5 (3/3)	3-4 (1/3)
	Inoculated CL[7]	0.67±0.14 (2/3)	0.67±0.14 (2/3)	0.58±0.14 (1/3)	ND (0/3)	ND (0/3)	ND (0/3)		
	Contacted OP	0.67±0.14 (2/3)	1.42±0.14 (3/3)	0.58±0.14 (1/3)	ND (0/3)	ND (0/3)	ND (0/3)	5-6 (3/3)	0-2 (0/3)
	Contacted CL	0.92±0.9 (3/3)	0.67±0.14 (2/3)	ND (0/3)	ND (0/3)	ND (0/3)	ND (0/3)		

[1]EID50, 50% embryo infective dose;
[2]DPI, days post infection;
[3]OP, oropharyngeal swab sample;
[4]The viral titers are shown as the mean ± SD from three separate experiments;
[5]ND, not detected;
[6]Positive birds / total birds sampled per time point;
[7] CL, cloacal swab sample.

Table S5. Virus shedding and antibody titers of contacted ducks through chickens inoculated with H9N2 virus.

Virus	Route	Virus titer (log_{10} EID_{50}[1]/0.1 mL, mean ± SD)	Seroconversion (log2)

		3 dpi[2]	5 dpi	7 dpi	9 dpi	11 dpi	13 dpi	14 dpi	21 dpi
CK/JS/1/2025(H9N2)	Contacted OP[3]	1.33±0.14[4] (3/3)[6]	1.25±0 (3/3)	0.58±0.14 (1/3)	ND[5] (0/3)	ND (0/3)	ND (0/3)	7-8 (3/3)	6 (3/3)
	Contacted CL[7]	0.92±0.29 (3/3)	1.33±0.14 (3/3)	ND (0/3)	ND (0/3)	ND (0/3)	ND (0/3)		

[1]EID_{50}, 50% embryo infective dose;

[2]DPI, days post infection;

[3]OP, oropharyngeal swab sample;

[4]The viral titers are shown as the mean ± SD from three separate experiments;

[5]ND, not detected;

[6] Positive birds / total birds sampled per time point;

[7] CL, cloacal swab sample.
